# Predictive potential of ACE phenotyping in extrapulmonary sarcoidosis

**DOI:** 10.1186/s12931-022-02145-z

**Published:** 2022-08-22

**Authors:** Sergei M. Danilov, Olga V. Kurilova, Valentin E. Sinitsyn, Armais A. Kamalov, Joe G. N. Garcia, Steven M. Dudek

**Affiliations:** 1 Division of Pulmonary, Critical Care, Sleep and Allergy, Department of Medicine, University of Illinois at Chicago, CSB 915, MC 719, 840 S. Wood Ave., Chicago, IL 60612 USA; 2 Medical Center, Moscow University, Moscow, Russia; 3 University of Arizona Health Sciences, Tucson, AZ USA

**Keywords:** Angiotensin I-converting enzyme, Plasma ACE, Systemic sarcoidosis, Conformational changes, Screening

## Abstract

Elevated ACE expression in tissues (reflected by blood ACE levels) is associated with increased risk of cardiovascular diseases and is also a marker for granulomatous diseases. We developed a new approach for characterization of ACE status in the blood—ACE phenotyping and established normal values of ACE levels 50–150% of control pooled plasma. ACE phenotyping was performed in citrated plasma of 120 patients with known interstitial lung diseases. In the 1st set of 100 patients we found 22 patients with ACE levels > 150%; ACE phenotyping also objectively identified the presence of ACE inhibitors in the plasma of 15 patients. After excluding these patients and patient with ACE mutation that increases ACE shedding, 17 patients were identified as a suspicious for systemic sarcoidosis based on elevation of blood ACE (> 150% of mean). A new parameter that we have established–ACE immunoreactivity (with mAb 9B9)—allowed us to detect 22 patients with decreased values (< 80%) of this parameter, which may indicate the presence of ACE in the blood that originates from macrophages/dendritic cells of granulomas. In the remaining 20 patients, this new parameter (mAbs binding/activity ratio) was calculated using 3 mAbs (9B9, 3A5 and i1A8—having overlapping epitopes), and 8 patients were identified as having decreases in this parameter, thus increasing dramatically the sensitivity for detection of patients with systemic sarcoidosis. Whole body PET scan confirmed extrapulmonary granulomas in some patients with lower immunoreactivity towards anti-ACE mAbs. ACE phenotyping has novel potential to noninvasively detect patients with systemic sarcoidosis.

**Supplementary Information:**

The online version contains supplementary material available at 10.1186/s12931-022-02145-z.

## Introduction

Sarcoidosis is a systemic inflammatory disease that occurs throughout the world and affects people of all races and ages. Despite years of study and recent advances in diagnostic strategies, the etiology of sarcoidosis and many details of the pathogenesis remain unknown [1–5].

Serum levels of angiotensin I-converting enzyme (ACE or CD143) are elevated in many patients with sarcoidosis and initially were thought to parallel the activity of sarcoid lesions [6–9]. However, this initial excitement about serum ACE representing a useful biomarker for sarcoid activity has proven to be incorrect. Multiple studies have demonstrated that serum ACE levels do not accurately reflect disease activity, and no correlation has been observed between serum ACE and ACE levels in granulomas [7, 10–12].

ACE is a Zn^2+^ carboxydipeptidase which is vital for the regulation of blood pressure and also associated with the development of vascular pathology. ACE is constitutively expressed on the surface of endothelial cells, absorptive epithelial and neuroepithelial cells, and cells of the immune system (macrophages, dendritic cells). Blood ACE likely originates from endothelial cell ACE, primarily lung capillary endothelium, due to proteolytic cleavage by a still unidentified ACE secretase-see for review [13, 14].

ACE tissue expression and blood ACE levels are strongly influenced by genetic factors. The best-described is the ACE I/D polymorphism: the absence (deletion, D) rather than the presence (insertion, I) of 287 base marker (Alu repeat) is associated with significantly higher circulating ACE levels [15], as well as with ACE in lymphocytes [16], and in human cardiac tissues [17]. Despite the fact that this particular ACE I/D polymorphism accounts for only 20% of the total variation of tissue and serum ACE [18, 19], it allows for the interpretation of ACE activity in sarcoid patients more precisely by establishing three reference intervals for each genotype [20–23]. We have proposed that measuring the ACE level (ACE phenotyping) rather than ACE genotyping will be more informative for many purposes, including identification of associations of ACE levels with cardiovascular complications [19, 24, 25], as well as for diagnostic evaluation of sarcoidosis.

During 30 years of intensive study of the ACE protein, we have generated a unique set of monoclonal antibodies (mAbs) to more than 40 different epitopes on the N- and C-domains of human ACE [26–28]. Localization of the epitopes of all these mAbs on the ACE molecule (epitope mapping) have allowed us to identify and map critical functional regions of this essential enzyme and to establish the novel approach for ACE studies—conformational fingerprinting of ACE [26].

We also demonstrated that local conformation of ACE is organ- and tissue-specific due to differential glycosylation of the ACE protein [26, 29–31]. More specifically, this unique set of mAbs can help to distinguish ACE origin from different organs or tissues.

Since some reports indicate that sarcoidosis is increasing in frequency [32], possibly due to industrial contamination, it is likely that accurate quantitative determination of ACE in the blood will be even more clinically useful. Given that the lung (the primary organ for sarcoidosis involvement) has relatively tighter endothelial cells junctions in its capillaries, we have hypothesized that elevated blood ACE originates primarily from extrapulmonary granulomas residing in organs with fenestrated or discontinuous capillaries [26]. Therefore, elevated serum levels of ACE are more likely to reflect extrapulmonary sarcoidosis involvement. We have established a novel approach, blood ACE phenotyping, for the purpose of full characterization of ACE in plasma or serum [33–36].

In the current study, we applied this approach to characterize ACE status in patients with interstitial lung diseases and identified patients who have ACE in their blood originating not only from endothelial capillaries (mostly lung), as in healthy individuals, but also ACE from other sources (macrophages and dendritic cells of granulomas). Therefore, we believe that our method allows for the noninvasive detection of patients with systemic sarcoidosis.

## Materials and methods

### Chemicals

ACE substrates, benzyloxycarbonyl-l-phenylalanyl-l-histidyl-l-leucine (Z-Phe-His-Leu) and hippuryl-l-histidyl-l-leucine (Hip-His-Leu), were purchased from Bachem Bioscience Inc. (King of Prussia, PA, USA) and Sigma (St. Louis, MO, USA). Other reagents were obtained from Sigma (St. Louis, MO, USA). Enalaprilat was provided by Apotex (Toronto, Canada).

### Antibodies

Antibodies to human ACE used in this study include a set of mouse monoclonal antibodies to human ACE that have been previously described [26, 37], which recognize native conformations of the N and C domains of human ACE.

### Study participants

The study was approved by the Ethic Committees of the Medical Center of Moscow University, Tareev Clinic of Nephrology and Internal and Occupational Diseases, Sechenov Medical University, Moscow, Russia [38]. All corresponding procedures were carried out in accordance with institutional guidelines and the Code of Ethics of the World Medical Association (Declaration of Helsinki). All patients provided written informed consent to have serum and citrated plasma for ACE characterization.

### ACE activity assay

ACE activity in serum or citrated plasma preparations was measured using a fluorimetric assay with two ACE substrates, 2 mM Z-Phe-His-Leu or 5 mM Hip-His-Leu [34, 37, 39]. Briefly, 20 µl aliquots of serum or plasma (diluted 1/5 in PBS) were added to 96 well microplate with conical wells and then 100 µl of ACE substrate was added and incubated for the appropriate time at 37 °C (usually 1 h), after that 25 µl of 1.4 N NaOH was added to stop the enzymatic reaction and to increase pH. Then 25 µl of orthophthaldialdehyde (3.3. mg/ml in methanol or ethanol) was added for 37 °C for complexing with His-Leu (product of enzymatic reaction). After 10 min this reaction was stopped by 25 µl of 2.1 N NaOH. The protein pellet was formed, which was sedimented by centrifugation of the plate at 2000*g* for 2 min. The adduct (complex of His–Leu with OPD) was quantified fluorometrically (excitation 365 nM and emission 500 nm) directly in wells of microtiter plate. ACE activity in individual patients was expressed as % from pooled plasma/serum (control) collected from sera of healthy donors and purchased from Interstate Blood Bank, Inc. (Memphis, TN, USA), or prepared from citrated plasma of multiple donors (Medical Center of Moscow University). Before pooling, each plasma sample was preliminary tested for the presence of ACE inhibitors or conformationally changed ACEs, as described before [33]. Calculation of ZPHL/HHL ratio [39] was performed by dividing fluorescence of the sample with substrate ZPHL to that with substrate HHL.

### Immunological characterization of the blood ACE (plate immunoprecipitation assay)

Microtiter (96-well) plates (Corning, Corning, NY, USA) were coated with anti-ACE mAbs via goat anti-mouse IgG (Pierce, Rockford, IL, USA) bridge and incubated with 50 µl of plasma/serum samples at 1/5 dilution in PBS. After washing of unbound ACE, plate-bound ACE activity was measured by adding a substrate for ACE (usually Z-Phe-His-Leu) directly into the wells [19, 37]. The level of ACE immunoreactive protein, using high affinity mAb 9B9, was quantified as described previously [19]. Conformational fingerprinting of blood ACE with mAbs to ACE was performed and presented as described previously [26, 35].

### *WholebodyPETscan*

18F-fluorodeoxyglucose (18F-FDG) positron-emission tomography/computed tomography (FDG PET/CT)-whole-body PET/CT imaging was performed with the GE Discovery PET/CT 610 (Milwaukee, USA) scanner using the standard protocol. 355–510 MBq of FDG (according to the patient’s body weight) were injected into patients 60 min before the start of scanning. A whole-body CT scan was performed without and with intravenous contrast administration with tube current settings 130 kV, 50 mAs, a pitch of 1.5, a slice thickness of 5 mm, and a field of view of 70 cm. A PET scan was performed immediately after an unenhanced CT scan with a 3-min acquisition per bed position.

### Statistical methods

Statistical analysis was carried out using SPSS Statistics software (IBM, Chicago, IL, USA). Parametric data was compared with Student’s T-test, and Mann–Whitney U-test was used for nonparametric data. Differences in data sets were considered statistically significant at p < 0.05.

## Results and discussion

### Blood ACE phenotyping

Previously we developed a new approach to characterize blood ACE in individual patients–blood ACE phenotyping [33–36]. This approach includes not just determination of ACE activity (with two substrates, ZPHL and HHL), but also determination of a novel kinetic parameter, the ratio of the rates of the hydrolysis of these two substrates (ZPHL/HHL ratio), which is able to control for the native state of the N and C domains of ACE active centers and to reveal the potentially complicating presence of ACE inhibitors [33–36, 39]. The third parameter is the concentration of ACE immunoreactive protein [19], and finally, the fourth and most sensitive approach is conformational fingerprinting of ACE using a set of anti-ACE mAbs showing subtle conformational changes in ACE surface topography [26, 33–35, 42].

Only 60% of patients with histologically proven pulmonary sarcoidosis have an elevated ACE level in the blood [7]. We [26] and others [6, 40] hypothesized that this group of patients with elevated blood ACE are more likely to have systemic sarcoidosis. Recently, we performed ACE phenotyping in 300 sera samples from individuals without known sarcoidosis or other pulmonary disease. Our results revealed substantial inter-individual variability of ACE levels (activity and level of immunoreactive ACE protein) characterized by a threefold difference, i.e. indicating that ACE levels in this normal population can be characterized as 100 + 25% (variability from 50 to 150%) [36].

Based on these data and analysis of the existing literature, we propose that patients with elevated ACE activity (> 150%) may have systemic sarcoidosis.

In the current study, we performed ACE phenotyping in citrated plasma from 120 patients recruited from pulmonary clinics (Table 1) in order to characterize their ACE profiles more comprehensively (ACE activity, the kinetic characteristics, the amount of ACE immunoreactive protein, and ACE conformation). Sarcoidosis was diagnosed in 59 patients based upon histopathologic (morphologic) assessment of biopsy samples. In another 30 patients no biopsies were available, but the diagnosis of presumptive sarcoidosis was made based upon a combined assessment of clinical criteria, including chest CT imaging, whole body PET-CT, laboratory results, clinical manifestations, and course of the disease. At the time the plasma samples used in this study were obtained, these patients were being evaluated for the first time in the pulmonary referral clinic. None were receiving steroids or other immunosuppressant therapy at the time the samples were collected.

**Table 1:** Patient characteristics

Sarcoidosis	(n = 89)	%	Other lung diseases	(n = 31)	%
Diagnosis (morphologically/clinically)	59/30				
Gender, male/female	30/59	**33.7/66.3**	Gender, male/female	13/18	**41.9/58.1**
Age, years	**48.7** (23–73)		Age, years	**58.4** (27–88)	
Male age, years	**40.6** (23–65)		Male age, years	**56.8** (27–74)	
Female age, years	**53.1** (25–73)		Female age, years	**59.6** (32–88)	
Organs involved:		**%**	Disease:		**%**
Lymph node	81	**91.0**	ILD	18	**58.1**
Lung	80	**89.9**	SjS	5	**16.1**
Skin	20	**22.5**	Chronic bronchitis	5	**16.1**
Spleen	12	**13.5**	IgG4-AD	2	**6.5**
CNS	11	**12.4**	Systemic scleroderma	1	**3.2**
Kidney	9	**10.1**	Allergic alveolitis	1	**3.2**
Eye	9	**10.1**	Amyloidosis	1	**3.2**
Heart	8	**9.0**	Bronchial asthma	1	**3.2**
Joint	7	**7.9**	ACE inhibitor use	0	0
Liver	5	**5.6**			
Muscle	2	**2.2**			
ACE inhibitor use	11	**12.4**			

Bold values are mean values

Biopsies samples were available (and performed) from 59 patients. Other 30 patients obtained a diagnosis based on combined clinical criteria, such as results of chest CT, whole body PET-CT, laboratory tests, clinical manifestations and course of the disease. Age is shown as mean (extremes in range). Data are shown as mean (extremes in range) and number (%)

*ILD* interstitial lung disease, *SjS *Sjögren syndrome, *IgG4-AD* immunoglobulin G4-associated disease. Clinical characteristics of these patients are described in details in [38]

**Fig. 1:**
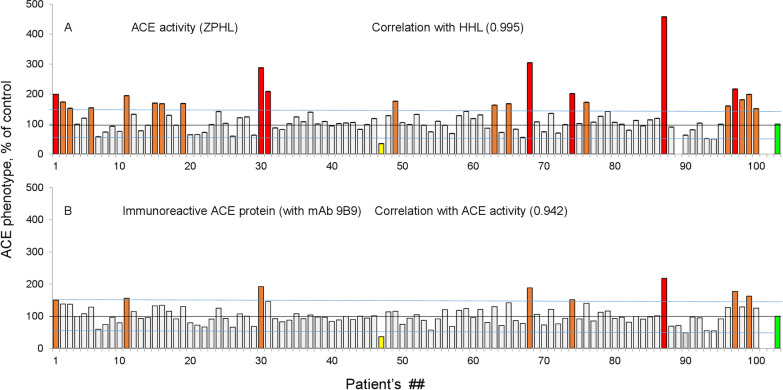
ACE levels in patient samples. Citrated plasma samples from 100 pulmonary clinic patients were analyzed for ACE activity using ZPHL substrate (**A**) and immunoreactive ACE protein levels using mAb 9B9 (**B**). Data are expressed as % of parameters of ACE levels (activity or the amount of immunoreactive ACE protein from value for control pooled plasma samples from healthy controls (green bars)). Bars with statistically significant changes in % of ACE precipitation are colored as follows: increase more than 20% (orange), > 50% (brown), more than twofold (red), decrease more than 50% (yellow). Shown are mean values from 2 to 5 experiments (each made in triplicates); SD (not shown for clarity) was less than 10%. Correlation coefficients are displayed in the figure

Figure 1A demonstrates ACE activity quantification in 100 patients with substrate Z-Phe-His-Leu (ZPHL). As a control (100%), we used pooled samples of citrated plasma from 3 blood donors. We found (1) 15 patients with ACE levels > 150% (highlighted in brown); compared to 6 such patients in a healthy population; (2) and 7 patients with 200% of ACE (red color)—compared to 0 in a healthy population. According to our preliminary hypothesis, these patients with ACE activity > 150% may have systemic sarcoidosis. Figure 1B demonstrates the precipitation of ACE activity from patients’ plasma by mAbs 9B9—there were 7 patients with > 150% the amount of immunoreactive ACE protein (with mAb 9B9); compared to 2 in the healthy population. 1 patient had ACE protein > 200% (red color)—compared to 0 in the healthy population. The correlation coefficient of ACE activity determination with the amount of ACE immunoreactive protein (with mAb 9B9) is extremely high at 0.942, which indicates a very high accuracy of measurement of ACE levels by both methods. Patient #87 has extremely high levels of both ACE activity and immunoreactive protein, and based on our experience, it is likely that this patient is a carrier of an ACE mutation leading to the increase in ACE shedding. Previously, we found several such mutations that result in increased rates of ACE shedding by different mechanisms, reviewed in [41]. In the same paper, we confirmed this suggestion and identified that patient #87 has a novel ACE mutation that increases ACE shedding through introduction of a stop codon (Q1224X) that eliminates its transmembrane anchor. As a result, ACE produced by one allele is directly secreted into circulation [41]. This patient was excluded from further analysis.

**Fig. 2:**
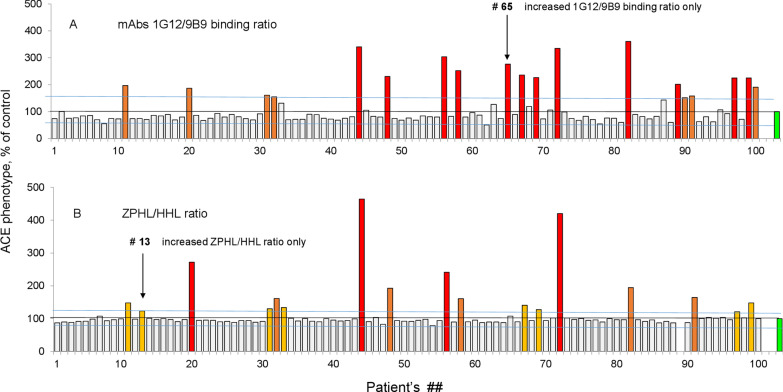
Identification of ACE inhibitor as a confounder in patient samples. Citrated plasma samples from 100 pulmonary clinic patients were analyzed for the presence of ACE inhibitor (ACEI) using the ZPHL/HHL ratio (**A**) and mAbs 1G12/9B9 binding ratio (**B**). Data are expressed as % of these parameters compared to those of control pooled plasma samples from healthy controls (green bars). Bar color scheme is as described in Fig. 1. Shown are mean values from 2 to 5 experiments (each made in triplicates); SD (not shown for clarity) was less than 10%. Concordance of these two parameters confirms the presence of ACEI in the samples

Subtle conformational changes in ACE can be detected in naïve plasma if concomitant ACE inhibitor use (ACEI) is not present. Because ACEI significantly alter the local conformation of ACE [33, 42], the detection of patients with ACE inhibitors in their blood (and further exclusion of them from consideration when using this technique to assess for the presence of systemic sarcoidosis) is an obligate step in ACE phenotyping. We have developed two independent and very sensitive methods for the detection of ACE inhibitors in the blood of patients. Figure 2A demonstrates the 1G12/9B9 binding ratio—the most sensitive parameter for the detection of exogenous ACEI in the blood [33, 35, 43]. We identified 19 patients with an elevated 1G12/9B9 ratio, suggesting likely ACE inhibitor usage.

Figure 2B demonstrates the kinetic parameter ZPHL/HHL ratio, which is significantly increased in the presence of ACE inhibitors [39]. We found 17 patients with elevated ZPHL/HHL ratios. However, the comparison of the 1G12/9B9 binding ratio (Fig. 2A) and ZPHL/HHL ratio (Fig. 2B) demonstrated clear concordance in only 15 patients—i.e., those in which both the 1G12/9B9 and ZPHL/HHL ratios are elevated. These 15 patients were confirmed to be taking ACE inhibitors, whereas in the case of elevation of only one parameter we likely are detecting different ACE mutations. For patient #13 (with elevated ZPHL/HHL ratio only), these are likely mutations in the active centers of N or C domains (similar to ACE mutation S333W) [44], and for patient #65, there likely are conformational changes in the N domain of ACE (similar to patients with uremia—see reference [33]). Therefore, only a combination of the two approaches allows for the unequivocal identification of patients taking ACE inhibitors.

### Detection of non-endothelial ACE in patient plasma.

The comparison of ACE activity in the plasma (Fig. 1A) and the amount of ACE immunoreactive protein precipitated by mAb 9B9 (Fig. 1B) clearly demonstrated that there are more patients with increased ACE activity than there are with elevated immunoreactive ACE protein. This observation suggests either the presence of unknown ACE activators, or diminishing of ACE inhibitor effects. Therefore, we next compared ACE activity and ACE immunoreactivity toward mAb 9B9.

**Fig. 3:**
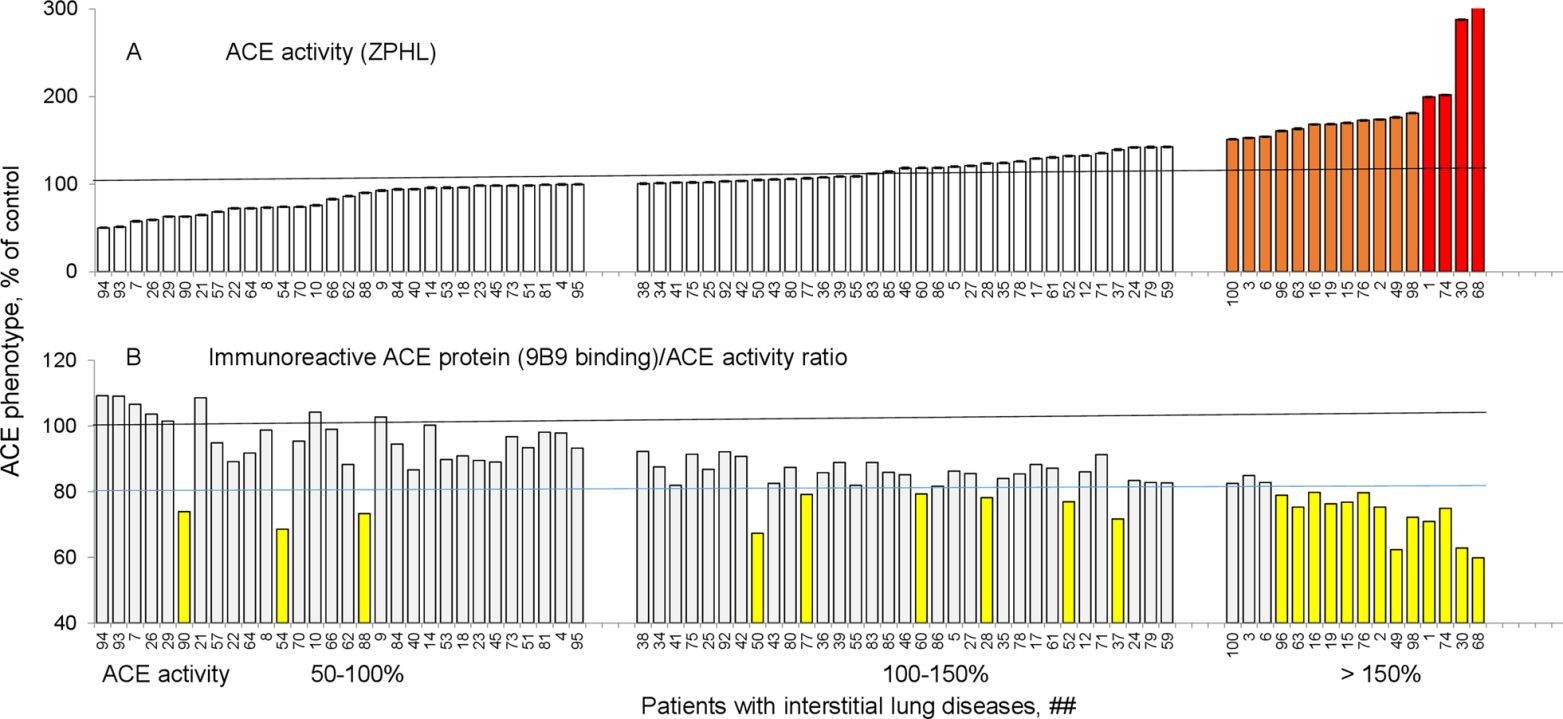
Utility of a new parameter—the ratio of immunoreactive ACE protein (with mAb 9B9) to ACE activity. Plasma samples from 100 pulmonary patients were analyzed for ACE activity using ZPHL substrate, with results displayed in ascending order (**A**). The ratio of ACE immunoreactive protein (with mAb 9B9) to ACE activity was then calculated (**B**), identifying the likely presence of non-endothelial-derived ACE in some plasma samples (yellow bars)

Figure 3A demonstrates ACE activity in patients (after excluding patients with ACEI in their blood (Fig. 2) and patient #87-with the novel ACE mutation dramatically increasing blood ACE levels) in increasing order, where samples with ACE activity > 150% are highlighted with brown and those > 200% are indicated with red. Figure 3B demonstrates a newly established parameter—the ratio of immunoreactive ACE protein (with mAb 9B9) to ACE activity-immunoreactivity of ACE towards mAb 9B9. The threshold for this parameter is set at 80% from control for this comparison. We hypothesized that this parameter discriminates blood ACE from healthy individuals (shed from endothelial cells-mainly lung capillaries) with ACE from patients with systemic sarcoidosis (which is a mixture of ACE from endothelial cells and ACE from activated macrophages/dendritic cells from sarcoid granulomas (yellow bars in Fig. 3B). The major advantage of this new parameter is that it allows for the identification of patients with systemic sarcoidosis among those with low baseline ACE levels (which is genetically determined), who otherwise could be missed if only our voluntary threshold of 150% ACE activity was used (since ACE levels vary from 50 to 150% of the general population mean). Decreased binding of ACE from granulomas to mAb 9B9 (for example due to differences in glycosylation of ACE in macrophages and ACE in endothelial cells) suggests that the decrease in 9B9 binding/activity ratio in patients with systemic sarcoidosis is likely not due to endogenous ACE inhibitors specific for patients with sarcoidosis [45]. Alternatively, it is more likely due to conformational changes in ACE from macrophages in granulomas (due to differential ACE glycosylation compared to endothelial cells).

**Fig. 4:**
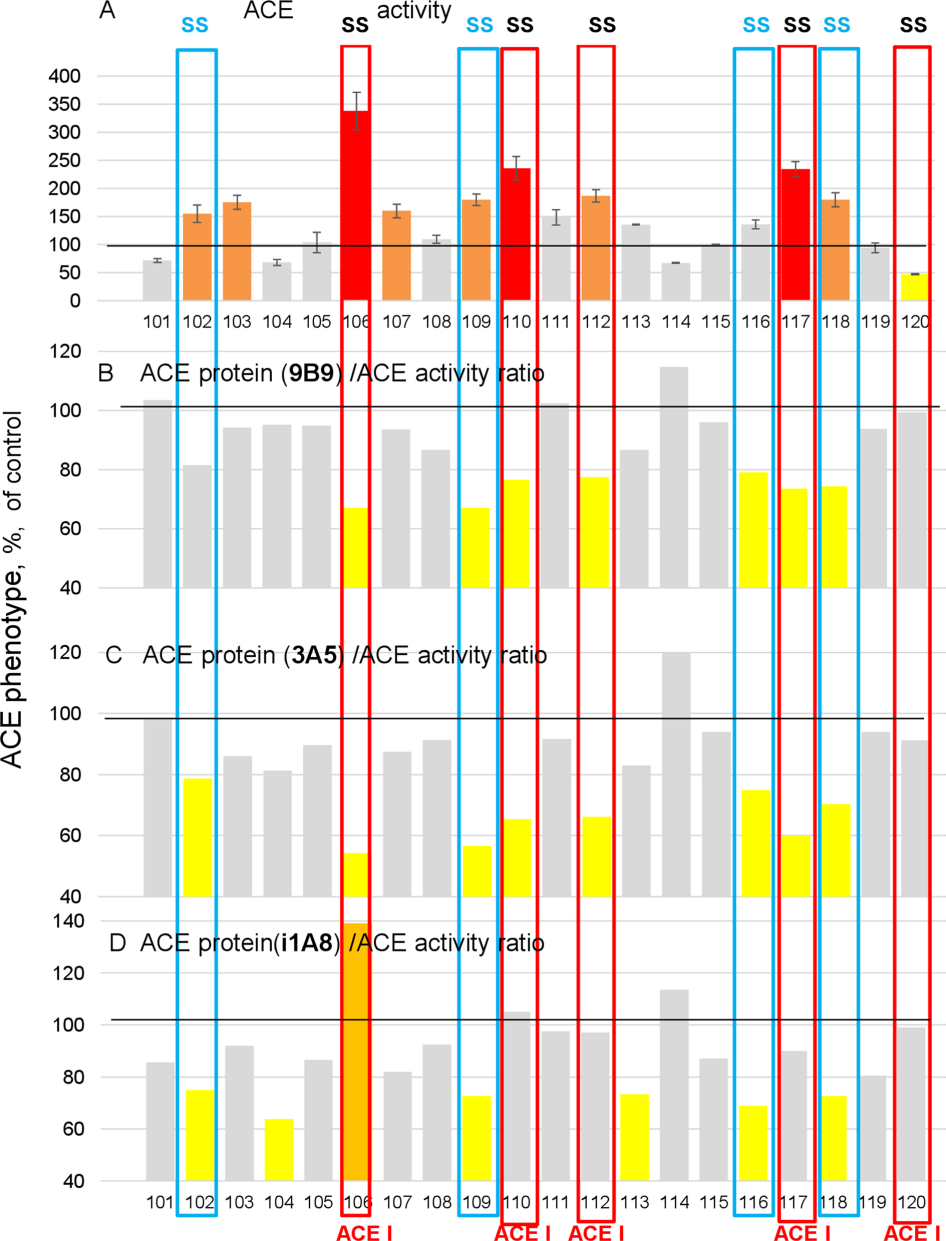
The ratios of immunoreactive ACE protein (using multiple mAbs) to ACE activity. Plasma samples from 20 pulmonary patients were analyzed for ACE activity using ZPHL as a substrate (**A**). The amount of immunoreactive protein ACE protein with mAb 9B9 (**B**), 3A5 (**C**) and i1A8 (**D**) was divided by mean ACE activity values for both substrates. Bar color scheme is as described in Fig. 1. Data are expressed as % of ACE levels (with different mAbs)/ACE activity ratio from value for control pooled sera samples from healthy controls. Mean values from 2 experiments (each made in triplicates); SD (not shown for clarity) was less than 10%. Patients that have decreased ratios for each mAb (less than 80% of control plasma) were marked with yellow. Patients having decreased ratio for all three mAbs were marked with blue. Patients that were taking ACE inhibitors (revealed as in Fig. 2) were marked with red

**Fig. 5:**
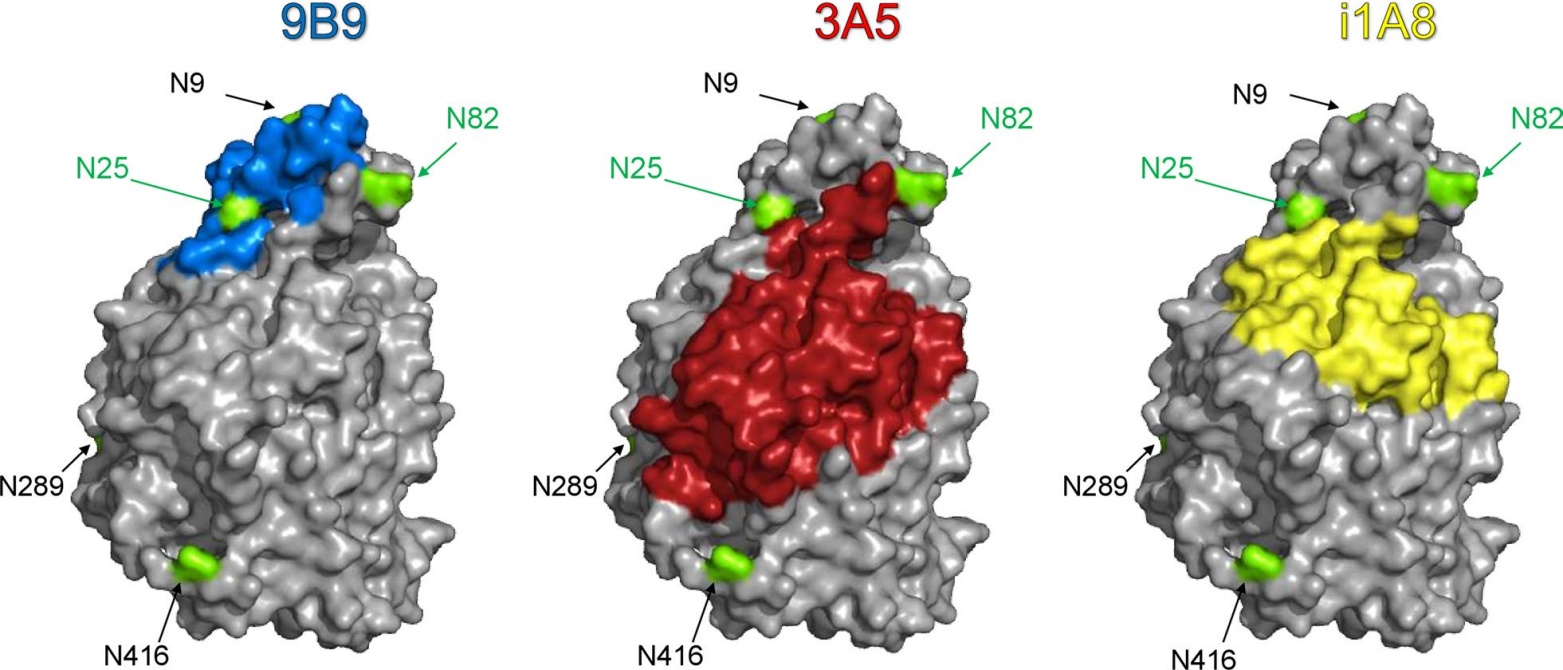
Epitope mapping of mAbs 9B9, 3A5, and i1A8. Surface representation of the N-domain of angiotensin-I-converting enzyme (ACE) based on the crystal structure 2C6N (Protein Database). Depicted is the structure of the N domain of ACE with marked potential glycosylation sites (by green), and epitope sites for mAbs 9B9, 3A5, i1A8 are colored in blue, red, and yellow respectively—based on [46]

A major limitation in the use of serum ACE measurements as a valid biomarker for sarcoidosis is the observation that ACE levels can be elevated in other disease processes. Our novel ACE fingerprinting approach helps address this problematic issue. For example, referring back to the data in Fig. 3B above, a potential confounding result could be obtained by any polymorphic variants influencing the ACE 9B9 epitope and changing the 9B9/activity ratio so that a patient was incorrectly assigned to the systemic sarcoidosis category. To address this possibility, we performed extended blood ACE phenotyping using a set of 6 mAbs in another 20 patients with suspected interstitial or sarcoidosis lung disease. These results (Fig. 4) further increased confidence in the potential for the ACE mAbs binding/activity ratio to identify patients with systemic sarcoidosis because four patients were identified (#102, #109, #116 and 118-blue boxed) in which the ACE mAbs binding/activity ratio was decreased for three of the mAbs tested (9B9, 3A5 and i1A8). These mAbs have overlapping epitopes, including Asn25 and Asn82 (Fig. 5), which are putative glycosylation sites on the N domain of ACE [46].

However, in patients taking ACE inhibitors this parameter could be applied only for two of these mAbs, because the presence of ACE inhibitors significantly increases mAb i1A8 binding to any ACE/ACE inhibitor complex (data not shown). This effect of ACE inhibitors is reflected in patients #106, #110, #112, 117, 120—red boxed in Fig. 4D. Therefore, we propose that the combination of the 9B9/activity ratio and 3A5/activity ratio (even in the presence of ACE inhibitors) when used together is optimal and has exciting potential as a novel blood assay for the identification of patients with systemic sarcoiosis.

### Conformational ACE fingerprinting identifies sarcoidosis “mimics”

The power of our ACE fingerprinting approach to identify sarcoidosis “mimics” is further illustrated by other examples. As noted above, high levels of blood ACE can be caused by certain ACE mutations that increase shedding of the enzyme or its secretion from endothelial cells (reviewed in [28]). While ACE mutations that eliminate transmembrane anchoring [47, 48] result in 14–20 fold increases in blood ACE and thus can be easily recognized, other ACE mutations that increase shedding and blood ACE levels by only four-sixfold [41, 42, 49, 50] are much more likely to be incorrectly attributed to possible sarcoidosis, leading to unnecessary diagnostic procedures or treatment for these patients [51]. One limitation of current clinical testing is that most laboratories do not determine the fold of ACE increase. We already have established an approach based on mAbs to the stalk region of ACE (the region in which ACE secretase cleaves ACE from the membrane) that can easily distinguish those with ACE mutations in this region from patients with sarcoidosis or Gaucher disease [52].

However, there are additional ACE mutations located outside the stalk region that nevertheless serve to increase ACE shedding by other mechanisms, such as changes in the degree of dimerization [50] or decreases in bilirubin binding, which normally fix ACE conformation on the membrane and prevent excessive ACE shedding [42]. With these types of mutations, our approach based on mAb 1B3 and 1B8 binding [52] does not distinguish between these subjects and those with sarcoidosis and Gaucher disease. Thus, additional approaches are needed. We recently described one such approach [41] that identified two more novel ACE mutations leading to dramatic increase in blood ACE levels. One of the main conclusions from this study [41] is that the frequency of such mutations is much higher than we thought previously, and thus the possibility to incorrectly diagnose sarcoidosis remains significant. Therefore, pulmonary physicians and other sarcoidosis specialists should be aware of this potential problem.

### Whole body PET scan to identify extrapulmonary granulomas

To summarize the potential of this approach for the 20 subjects described in Fig. 4, 8 patients (102, 106, 109, 110, 112, 117, 118) have elevated total ACE levels > 150%, suggestive of possible systemic sarcoidosis. In addition, 4 patients (blue boxed) exhibit > 20% decreases in the mAb binding/ACE activity ratios for 3 mAbs (9B9/3A5/i1A8)-, while 4 patients exhibit > 20% decreases in the mAb binding/ACE activity ratios for at least 2 mAbs (9B9/3A5). Thus, 8 of these 20 patients have ACE conformational fingerprint patterns that are suggestive of extrapulmonary sarcoidosis involvement (blue and red boxed) (except #120).

To confirm that elevated blood ACE levels, or decreased mAb binding/ACE activity ratios for some mAbs (9B9/3A5/i1A8), indicate association with systemic (extrapulmonary) sarcoidosis, we performed whole body PET scanning in selected patients in which ACE phenotyping was performed. In patient #82B (from another cohort of unrelated patents) with elevated total ACE levels (180%), but no reduction in the mAb binding/ACE activity ratios (data not shown), whole body PET scanning demonstrated no abnormal FDG uptake and no signs of lymph node enlargement (Additional file 1: Fig. S1). This patient did not have sarcoidosis but instead was found to have hyperthyroidism, which is known to increase tissue ACE expression and thus serum ACE activity [53]. This example demonstrates the potential of this approach to “rule out” systemic sarcoidosis despite the presence of an elevated ACE level. The potential to exclude extrapulmonary organ involvement with a relatively noninvasive and less costly test than PET scanning represents a significant clinical advance.

**Fig. 6:**
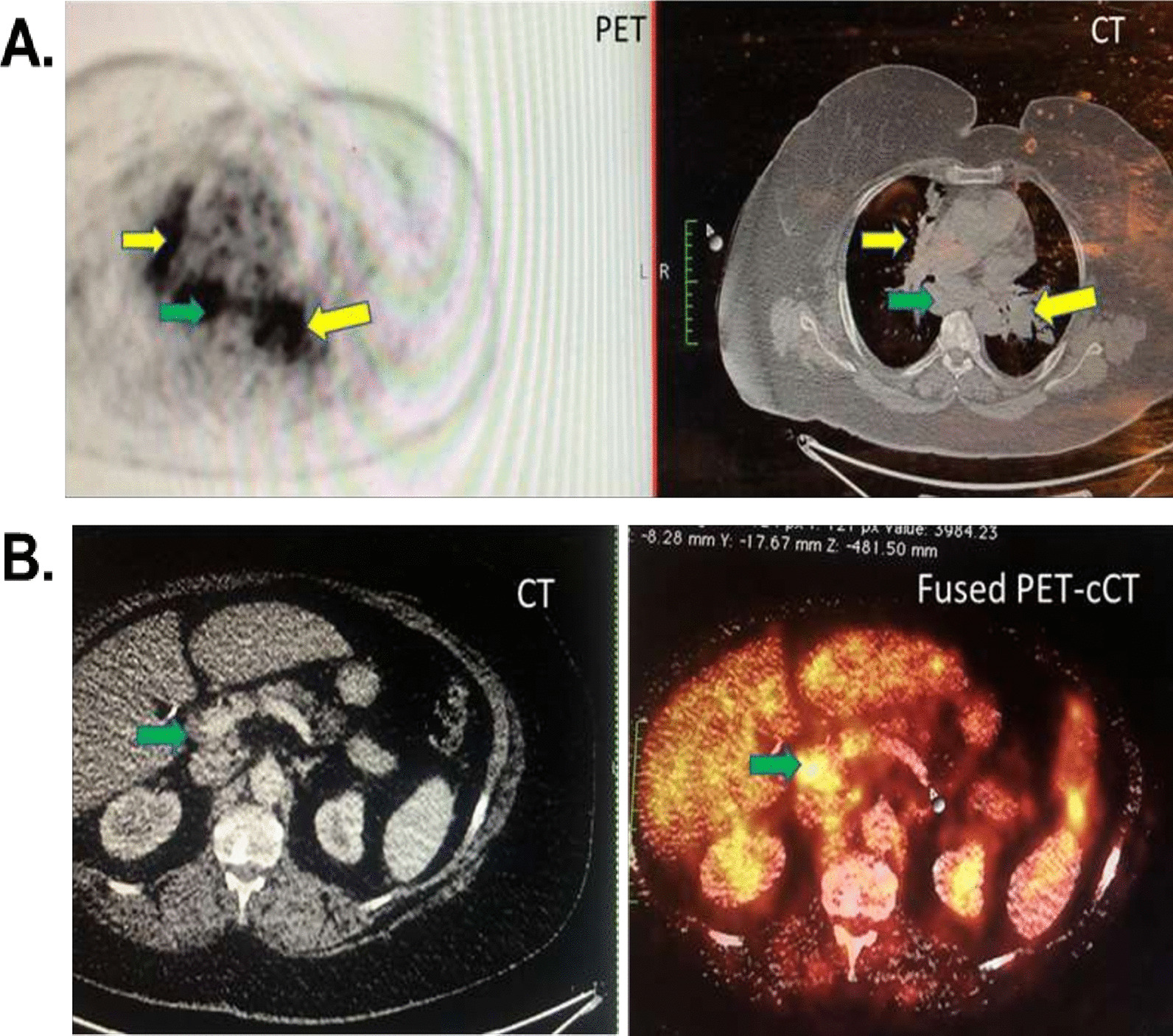
Whole body PET scan in patient #117 with suspected systemic sarcoidosis. Whole body PET/CT with FDG was performed on patient #117. Panel **A** illustrates PET (left side) and CT (right side) images from the same level within the chest. Yellow arrows indicate FDG uptake in hilar lymph nodes, and green arrows indicate FDG uptake in mediastinal nodes. Panel **B** illustrates CT (left side) and fused PET/CT (right side) images and demonstrate moderate increased FDG uptake in the liver and in the periportal lymph nodes (marked with green arrows)

**Fig. 7:**
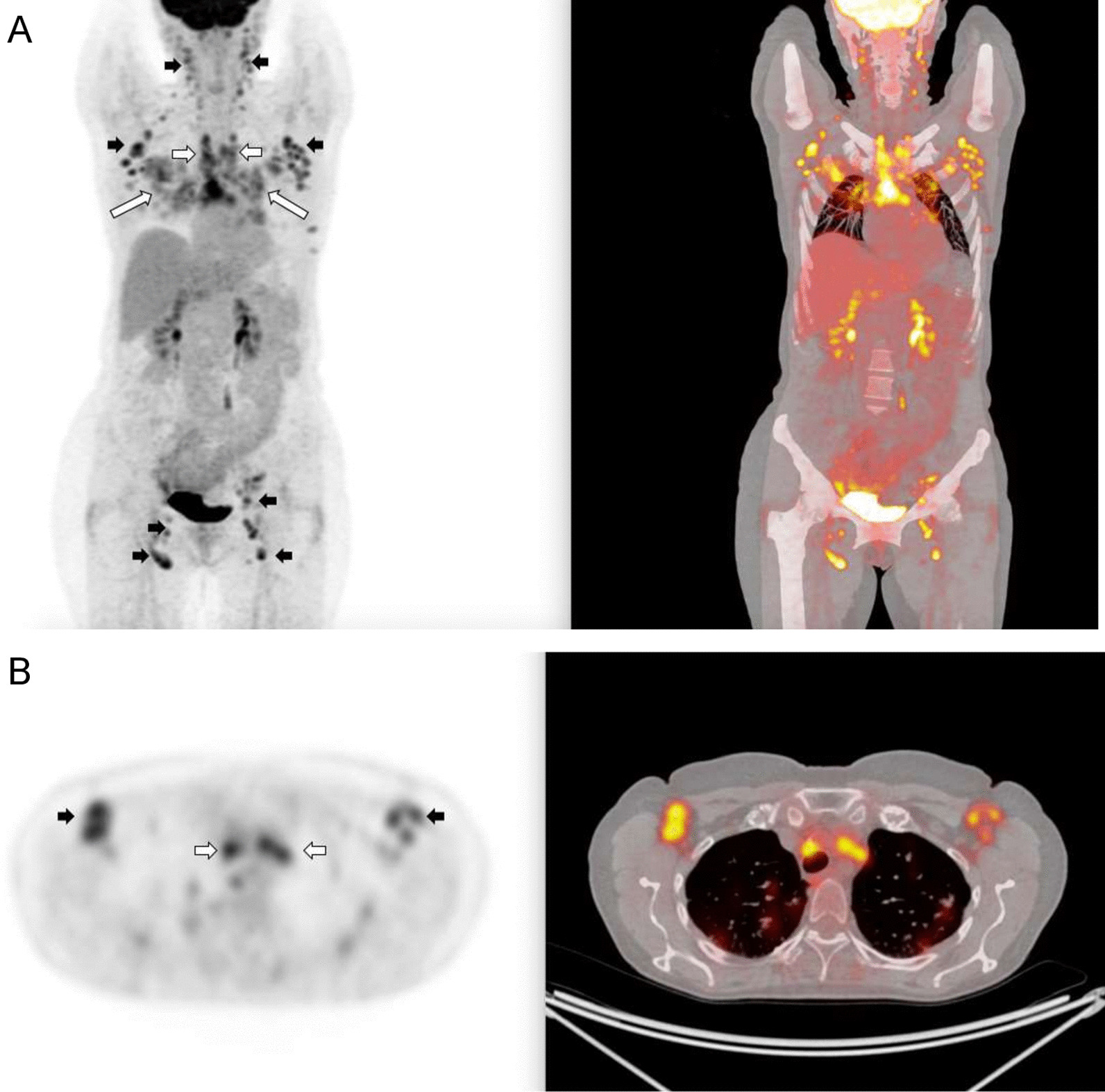
Whole body PET scan in patient #118 with suspected systemic sarcoidosis. Whole body PET/CT with FDG was performed on patient #118. **A** FDG PET maximum intensity coronal projection (left image) and FDG PET/CT fused coronal projection (right image). Images demonstrate a high FDG uptake in the mediastinal and hilar lymph nodes (indicated with the short white arrows) and nodules in the lung fields (long white arrows). There is a marked bilateral uptake of FDG in the extrapulmonary groups of axillary, supraclavicular, jugular and inguinal lymph nodes. These groups of lymph nodes are indicated with short black arrows. **B** FDG PET maximum intensity axial projection (left image) and FDG PET/CT fused axial projection (right image). Images demonstrate a high bilateral FDG uptake in the axillary lymph nodes (indicated with the short white arrows) and mediastinal nodules (short white arrows)

Whole body PET scanning next was performed on two patients with decreased mAb binding/ACE activity ratios (#117 and #118 from Fig. 4) as a further proof of concept. Figure 6 imaging (patient #117) demonstrated increased FDG uptake in the enlarged hilar/mediastinal lymph nodes, indicative of pulmonary involvement, as well as moderately increased FDG uptake in the liver, consistent with systemic sarcoidosis as predicted by our ACE fingerprinting approach. Whole body PET/CT performed on patient #118 (Fig. 7) also showed increased 18F-FDG-uptake in multiple mediastinal and hilar lymph nodes, reticular fibrosis and small perilymphatic nodules in the lung fields. In addition to this typical pattern of pulmonary and mediastinal sarcoidosis, PET/CT revealed prominent enlargement with high 18F-FDG uptake in multiple other major extrapulmonary groups of lymph nodes—in the neck, supraclavicular, axillary and inguinal regions (Fig. 7A, B).

### Statistical analysis of ACE phenotype in sarcoidosis groups

We next evaluated ACE activity in 68 patients with clinically verified sarcoidosis, which was significantly higher than in 298 unrelated patients—median (interquartile range): 118.7 (98.9–160.4) vs. 97.3 (81.7–117.5), p < 0.001, respectively) (Additional file 2: Fig. S2A). Our newly established parameter mAb 9B9 binding/ACE activity ratio also demonstrated a statistically significant decrease in sarcoidosis patients compared to healthy individuals (99.9 (91.5–112.4) vs. 88.9 (81.3–94.7), p < 0.001, respectively) (Additional file 2: Fig. S2B). However, neither ACE activity nor 9B9 binding/activity ratio were significantly different in 37 patients with clinically defined extrapulmonary sarcoidosis versus 31 sarcoidosis patients with only lung involvement (not shown). This absence of statistically significant differences between these 2 groups of sarcoidosis patients also has been observed previously-not only for ACE activity, but for serum chitotriosidase level as well [54]. Relevant to our hypothesis, increased ACE activity or decreased mAb 9B9 binding/activity ratio could occur when a proportion of excess ACE from sarcoid granulomas (i.e., granuloma load) becomes significantly elevated. The observation that 22 of 44 patients with clinically defined extrapulmonary sarcoidosis demonstrated decreased mAb 9B9 binding/activity ratio may indicate that only in these patients (50%) did the extrathoracic granuloma load reach a significant threshold.

## Conclusions

We performed complete ACE phenotyping in 120 plasma samples from pulmonary clinic patients with interstitial lung disease. Because we previously established a normal range of ACE levels (based on ACE phenotyping of 300 unrelated patients (healthy personnel), which varies threefold in the tested population: ranged between 50–150% from control pooled plasma, we found that 31 patients could be considered as possible candidates for systemic sarcoidosis based only on ACE levels. However, an application of conformational fingerprinting allowed us to identify another set of 22 patients based on another parameter of ACE phenotype (ratio of ACE immunoreactive protein (with mAb 9B9) to ACE activity.

Our approach incorporates multiple parameters to characterize serum ACE in clinically-relevant ways. These parameters include (1) combination of ACE activity determination with two substrates; (2) quantification of ACE immunoreactive ACE protein with a set of mAbs to ACE; and (3) estimation of ACE conformation in each individual.

To summarize the potential clinical importance of these parameters, the ACE fingerprinting approach provides a relatively noninvasive way through blood sampling to eliminate sarcoidosis from consideration in selected patients with elevated ACE levels, as well as detect the presence of sarcoidosis in patients in whom low total ACE levels (often genetically determined) may result in the appropriate diagnosis not being considered. It also provides information useful for identifying systemic sarcoidosis in some patients, thus preventing them from undergoing more invasive (e.g., biopsy) and/or expensive testing (e.g., PET). Because some extrapulmonary manifestations (especially cardiac and neurologic) are associated with higher mortality and/or more severe morbidity than pulmonary sarcoidosis alone, early detection is an important clinical goal. Thus, this comprehensive approach advances precision medicine in sarcoidosis by providing a noninvasive and relatively low cost method for detecting sarcoidosis patients with low total ACE levels, screening for mutations that result in markedly elevated ACE levels in the absence of sarcoidosis, and also identification of patients with systemic sarcoidosis involvement. As a result, the ACE phenotyping method has the potential add significant value very early in the evaluation process for patients with suspected sarcoidosis. If utility and accuracy is confirmed in additional studies, we propose it be added to the algorithm for sarcoidosis evaluation as early as the first clinical visit in which sarcoidosis is suspected.

## Supplementary Information

**Additional file 1: Fig.** **S1.** Unremarkable whole body PET scan in a patient with elevated blood ACE levels. A whole body PET/CT was performed on a patient (#82B) with elevated blood ACE level (180%). Top panels shows multi-planar PET reformations –sagittal (**A**), axial (**B**) and coronal (frontal) (**C**). Bottom panel illustrates fused PET/CT (**D**) and CT (**E**) images demonstrating no increased FDG uptake in the areas of mediastinal or hilar lymph nodes. Further examinations confirmed that the patient had hyperthyroidism rather than sarcoidosis.

**Additional file: 2: Fig.** **S2.** Comparison of ACE parameters in healthy individuals and sarcoidosis patients. Box-and-whisker plot for ACE activity (**A**). Box-and-whisker plot for mAb 9B9 binding/ACE activity ratio (**B).** Line inside the box—median; limits of the box—75th and 25th percentiles; whiskers—10th and 90^th^ percentiles; •—outliers. P values are displayed in the figure.

## Data Availability

All data generated or analysed during this study are included in this published article (and its Additional files).
