# Supplementary figures and images for: Predictive potential of ACE phenotyping in extrapulmonary sarcoidosis
Source: Respir Res. 2022 Aug 22;23:211. doi: 10.1186/s12931-022-02145-z (PMC9396819; doi:10.1186/s12931-022-02145-z)

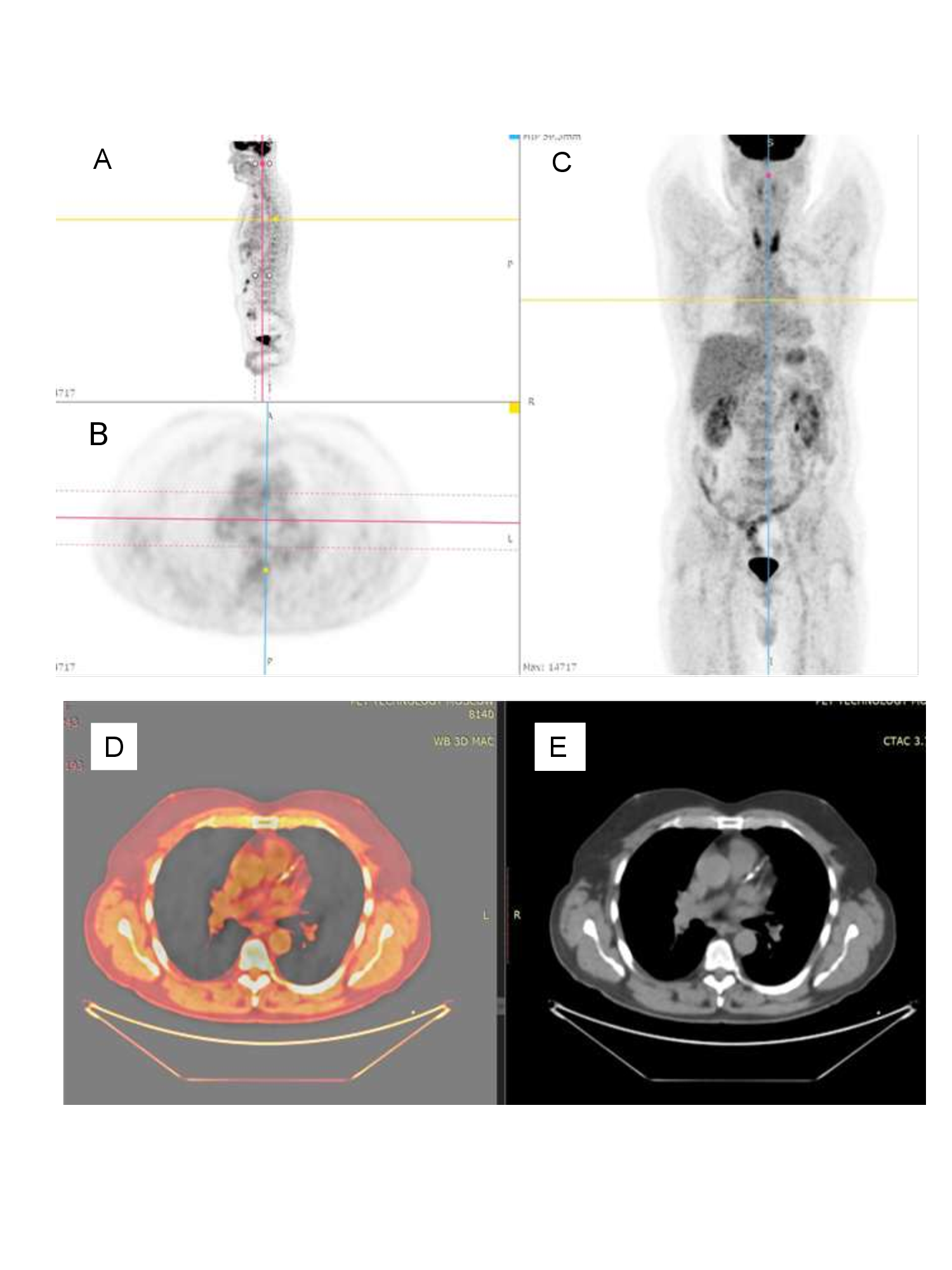

Supplement: Supplementary file 1 — Additional file 1: Fig. S1. Unremarkable whole body PET scan in a patient with elevated blood ACE levels. A whole body PET/CT was performed on a patient (#82B) with elevated blood ACE level (180%). Top panels shows multi-planar PET reformations –sagittal (A), axial (B) and coronal (frontal) (C). Bottom panel illustrates fused PET/CT (D) and CT (E) images demonstrating no increased FDG uptake in the areas of mediastinal or hilar lymph nodes. Further examinations confirmed that the patient had hyperthyroidism rather than sarcoidosis. [file 12931_2022_2145_MOESM1_ESM.tiff]

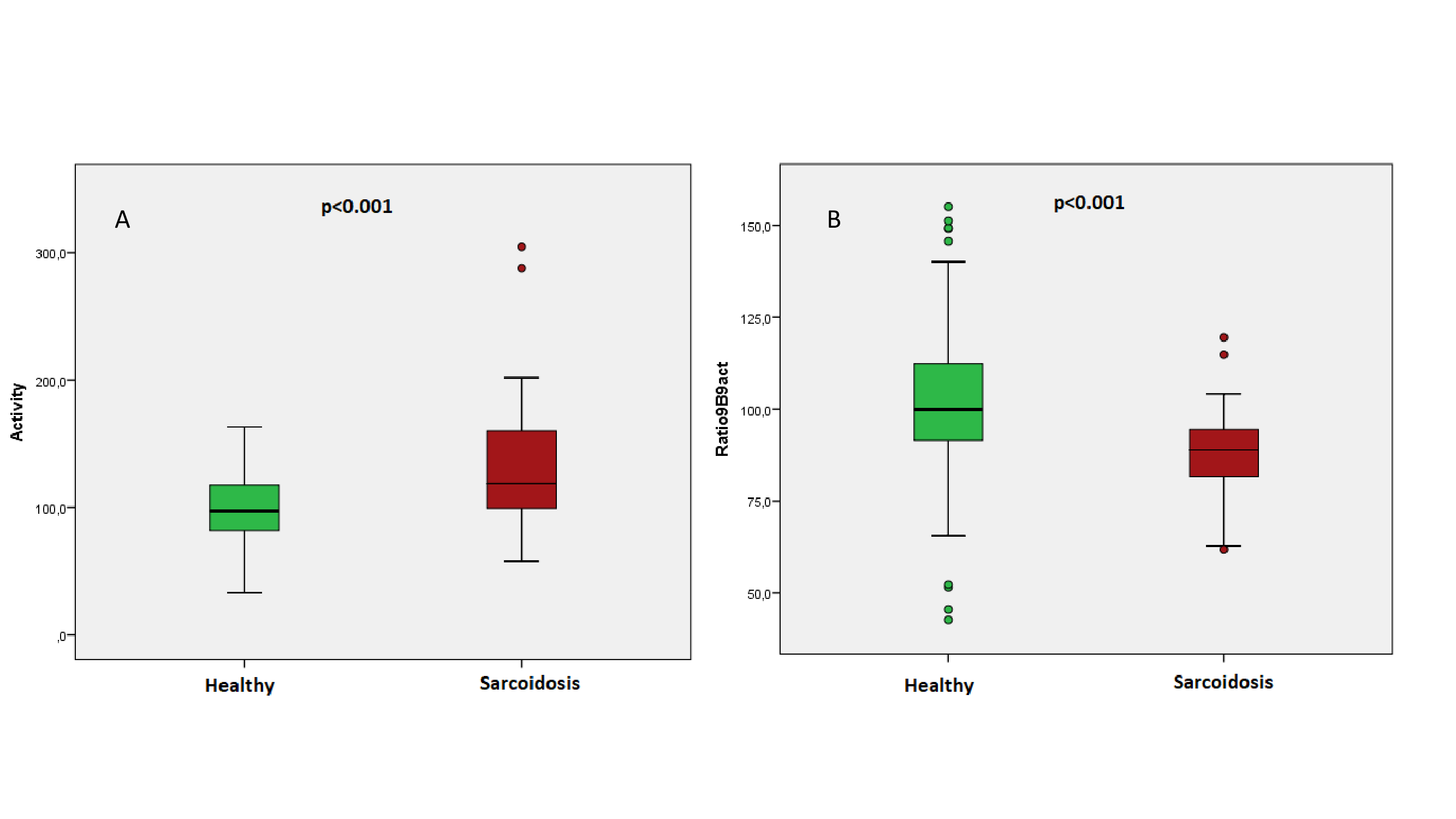

Supplement: Supplementary file 2 — Additional file: 2: Fig. S2. Comparison of ACE parameters in healthy individuals and sarcoidosis patients. Box-and-whisker plot for ACE activity (A). Box-and-whisker plot for mAb 9B9 binding/ACE activity ratio (B). Line inside the box—median; limits of the box—75th and 25th percentiles; whiskers—10th and 90th percentiles; •—outliers. P values are displayed in the figure. [file 12931_2022_2145_MOESM2_ESM.tiff]
